# Musical Auditory Alpha Wave Neurofeedback: Validation and Cognitive Perspectives

**DOI:** 10.1007/s10484-021-09507-1

**Published:** 2021-04-30

**Authors:** Kazuhiko Takabatake, Naoto Kunii, Hirofumi Nakatomi, Seijiro Shimada, Kei Yanai, Megumi Takasago, Nobuhito Saito

**Affiliations:** Department of Neurosurgery, The University of Tokyo, 7-3-1 Hongo, Bunkyo-ku, Tokyo, 113-8655 Japan

**Keywords:** Neurofeedback, Alpha wave, Wearable EEG, Short-term memory, Working memory

## Abstract

Neurofeedback through visual, auditory, or tactile sensations improves cognitive functions and alters the activities of daily living. However, some people, such as children and the elderly, have difficulty concentrating on neurofeedback for a long time. Constant stressless neurofeedback for a long time may be achieved with auditory neurofeedback using music. The primary purpose of this study was to clarify whether music-based auditory neurofeedback increases the power of the alpha wave in healthy subjects. During neurofeedback, white noise was superimposed on classical music, with the noise level inversely correlating with normalized alpha wave power. This was a single-blind, randomized control crossover trial in which 10 healthy subjects underwent, in an assigned order, normal and random feedback (NF and RF), either of which was at least 4 weeks long. Cognitive functions were evaluated before, between, and after each neurofeedback period. The secondary purpose was to assess neurofeedback-induced changes in cognitive functions. A crossover analysis showed that normalized alpha-power was significantly higher in NF than in RF; therefore, music-based auditory neurofeedback facilitated alpha wave induction. A composite category-based analysis of cognitive functions revealed greater improvements in short-term memory in subjects whose alpha-power increased in response to NF. The present study employed a long period of auditory alpha neurofeedback and achieved successful alpha wave induction and subsequent improvements in cognitive functions. Although this was a pilot study that validated a music-based alpha neurofeedback system for healthy subjects, the results obtained are encouraging for those with difficulty in concentrating on conventional alpha neurofeedback.

Trial registration: 2018077NI, date of registration: 2018/11/27

## Introduction

Neurofeedback is a method to self-regulate brain function by measuring brain activity and presenting it to subjects. Brain activity is assessed using various modalities, such as functional magnetic resonance imaging (fMRI) (Sulzer et al., 2013), near-infrared spectroscopy (NIRS) (Mihara et al., 2012), scalp electroencephalogram (EEG) (Zoefel et al., 2011), and intracranial EEG (Yamin et al., 2017). One of the most widely utilized methods of neurofeedback involves the alpha wave from EEG (Yeh et al., 2020).

The alpha wave is an 8–13 Hz brainwave that is mainly observed in the occipital region of healthy individuals and is enhanced by closing the eyes. The augmentation of alpha waves by neurofeedback may mitigate the symptoms of psychiatric disorders, such as anxiety and depression (Markiewcz, 2017; Schoenberg & David, 2014; Schönenberg et al., 2017). An increasing number of studies have reported the usefulness of alpha wave feedback in patients with attention-deficit and hyperactivity disorder (ADHD) and emphasized its importance in clinical settings (Lofthouse et al., 2012). However, some patients with ADHD cannot concentrate on alpha neurofeedback for an entire session, resulting in varying rates of drop-outs (Duric et al., 2012; Gevensleben et al., 2010). Previous studies mostly adopted a neurofeedback protocol in which the amplitude of alpha wave power was visually presented (Biswas & Ray, 2019; Choi et al., 2011; Escolano et al., 2014; Gruzelier, 2014; Hsueh et al., 2016; Lavy et al., 2019). Subjects need to spend the entire time concentrating on a monitor, which may be intolerable for some children and the elderly as well as patients with ADHD. Since the framework of alpha neurofeedback requires the continuous participation of subjects in repeated sessions, a novel protocol in which subjects may relax during the session needs to be developed.

Previous studies on auditory alpha neurofeedback reported the augmentation of the alpha wave as well as improvements in cognitive function (Bucho et al., 2019; Cho et al., 2008; Fernández et al., 2016; van Boxtel et al., 2012). However, these studies adopted monotonous auditory stimulations, which may not be relaxing subjects during neurofeedback sessions. The use of music may overcome this issue. Music has been used to improve various neurological conditions. Recently, biofeedback using sonification has been shown to be effective and is attracting a growing attention (Bergstrom et al., 2014; Brancatisano et al., 2020; Fedotchev et al., 2018; Maes et al., 2016). However, there are only a limited number of studies reporting that combining such music therapy and alpha neurofeedback has neuroprotective effect (Alexander, 2018; Nawaz et al., 2018; Ramirez et al., 2015). Prior to the introduction of music-based alpha neurofeedback to individuals with difficulty maintaining concentration, it is important to validate whether the protocol effectively induces the alpha wave and clarify its effects on cognitive functions in healthy subjects.

Therefore, in the present study, healthy subjects underwent more than two-month-long sessions of neurofeedback using ALPHA SWITCH ver.0.9.0 (Mediaseek Inc., Tokyo, Japan, https://www.mediaseek.co.jp/alpha-switch/, available on App Store), which is an application developed to provide an opportunity to experience auditory alpha-neurofeedback while listening to music. This system appears to free subjects from the stress of concentrating on a monitor with their eyes open, thereby reducing the burden on subjects to keep participating in long-term neurofeedback. This system is also almost free of EEG noises generated by eye movement and eye opening/closing, which is always a major issue in scalp EEG recordings because subjects are allowed to close their eyes during each session (Jebelli et al., 2018).

The primary purpose of the present study was to validate whether the novel auditory neurofeedback system augments the power of the alpha wave in healthy subjects. The secondary purpose was to compare changes in cognitive functions evaluated before, between, and after each feedback. The present study was a single-blind, randomized control crossover trial, which enables a more efficient comparison than a parallel design with fewer subjects because each subject serves as his/her own matched control.

## Methods

### Subjects

Subjects were required to be present for measurements in our facility for approximately 15 min per day for 3–4 days a week over two months. Ten doctors from the Department of Neurosurgery of the University of Tokyo participated in the present study. All subjects were male and the average age was 33.8 years (31–36 years).

No subjects had previously experienced neurofeedback, had taken any medications for the nervous system, or had a history of neurological or mental diseases. The present study was approved by the Research Ethics Committee, Graduate School of Medicine and Faculty of Medicine, the University of Tokyo. All subjects provided written informed consent.

### EEG Device

Muse (InteraXon Inc., Toronto, Canada, https://choosemuse.com/muse-2/) is a headband-type wearable EEG device that is mounted on the forehead with the end of the band held on each ear. Although EEG may be easily measured without a special pretreatment on the scalp with this device, we applied EEG paste on the scalp for recording in the present study. Muse has 4 active electrodes and one reference electrode. Two active electrodes made of silver chloride are located on the bilateral forehead and two other electrodes made of conductive silicon rubber are on the dorsal side of the bilateral auricles. The reference electrode is located between the two active electrodes on the forehead. The sampling rate was fixed at 256 Hz. Recorded data was transferred to the tablet device (iPad, Apple Inc., California, The United States of America) via Bluetooth without a delay. EEG data recorded by the four electrodes of Muse were saved in the respective four channels of the tablet device after being processed by a 50-Hz notch filter.

### Schedule of the Feedback Study

**Fig. 1:**
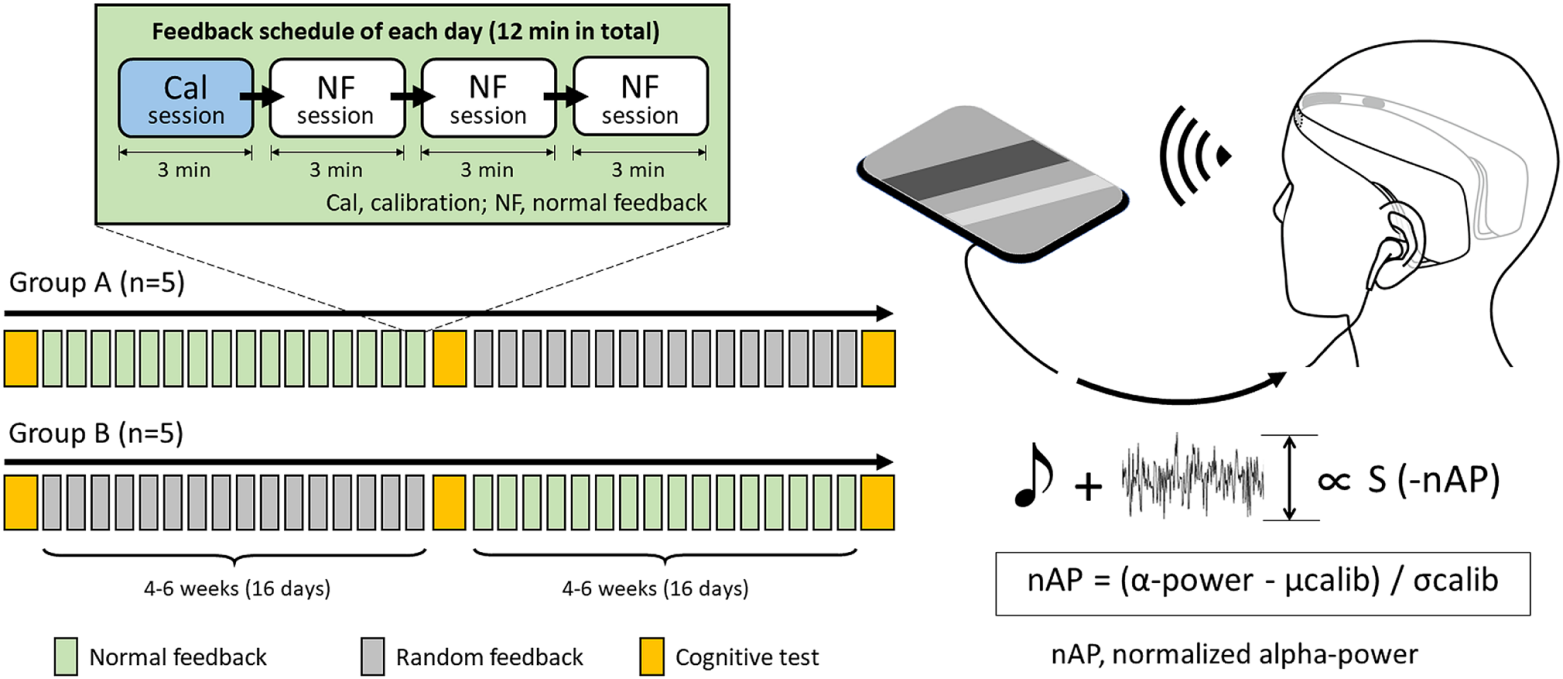
A schema of the schedule of neurofeedback in this study (left) and the adopted neurofeedback system (right). The subjects of group A underwent NF in the first feedback period and RF in the second feedback period, while subjects of group B underwent each feedback in reverse order. In the normal neurofeedback, the subject listened to classical music with the eyes closed and Muse mounted on the head. The superimposed white noise was updated every three seconds so that the noise level inversely correlated to the alpha-power, normalized to that of the calibration session. *NF* normal feedback, *RF* random feedback, *Cal* calibration, *α-power* averaged alpha power in feedback session for every three second, *μcalib* averaged alpha power in calibration session, *σcalib* standard deviation of alpha power in calibration session

The present study was designed as a crossover study, in which each subject went into the first and second feedback periods using normal feedback (NF) or random feedback (RF) in an assigned order (Fig. 1). Each subject was assigned to group A or B, each of which had 5 subjects. Subjects in group A underwent NF in the first feedback period and RF in the second feedback period, while subjects in group B received each feedback in the reverse order. The first and second feedback periods were separated by an interval of at least two days. Subjects were blind to which group they were assigned to and which feedback they were receiving.

In every feedback period, each subject was engaged in feedback sessions for three to four days a week depending on subject availability, resulting in each feedback period of 16 days ranging between four and six weeks. The subject performed one calibration session and three feedback sessions consecutively per day in a feedback period. We postponed the measurement when subjects felt too tired or drowsy to maintain their concentration for 15 min of feedback. In groups A and B, the cognitive functions of all subjects were evaluated three times; before, between, and after each feedback period.

### Neurofeedback

The subject was seated in a quiet room with their eyes closed and Muse mounted on the head. The recording and real time analysis of EEG and real time feedback of the analyzed result were performed using default function of ALPHA SWITCH as follows. Before the feedback session, EEG was recorded for 180 s for calibration, during which the reliability of the recording was checked by referring to the power law distribution of physiological EEG (Namazi & Kulish, 2012; van Albada & Robinson, 2013). Every three-second data set of each channel was processed by an 8–13-Hz bandpass filter and Hilbert transformation, yielding power in the alpha range (alpha-power). Averaged alpha-power was obtained every 3 s. Data was analyzed and output in real time by the application installed in the tablet device. Fast Fourier transformation was applied for every three seconds of EEG data and calculated the power spectral density curve. Using the least-squares method, the slope of the curve was calculated between 1 Hz and the Nyquist frequency. Three-second data with a slope between − 0.30 and − 0.14 were omitted due to excessive noise, and recordings were performed again when the omitted data surpassed 5% of all calibration data. The Smirnov-Grubbs test was used to exclude outliers (*p* < 0.05) from calibration data. The time average (μcalib) and standard deviation (σcalib) of alpha-power were calculated.

A feedback session for 180 s followed the calibration session. Durations of feedback sessions in the previous studies on alpha neurofeedback range between 3 and 30 min (Jirayucharoensak et al., 2019; Naas et al., 2019; Wei et al., 2017). We adopted this short duration in the present study to avoid sleeping during the feedback session with subjects’ eyes closed. Each subject underwent NF or RF according to the assignment. In the same manner as the calibration session, the time average of alpha-power was calculated every three seconds, and was then converted to z-scores of alpha-power using μcalib and σcalib. The normalized alpha-power (nAP) of each three-second data was obtained by averaging the z-scores of four channels.

**Fig. 2:**
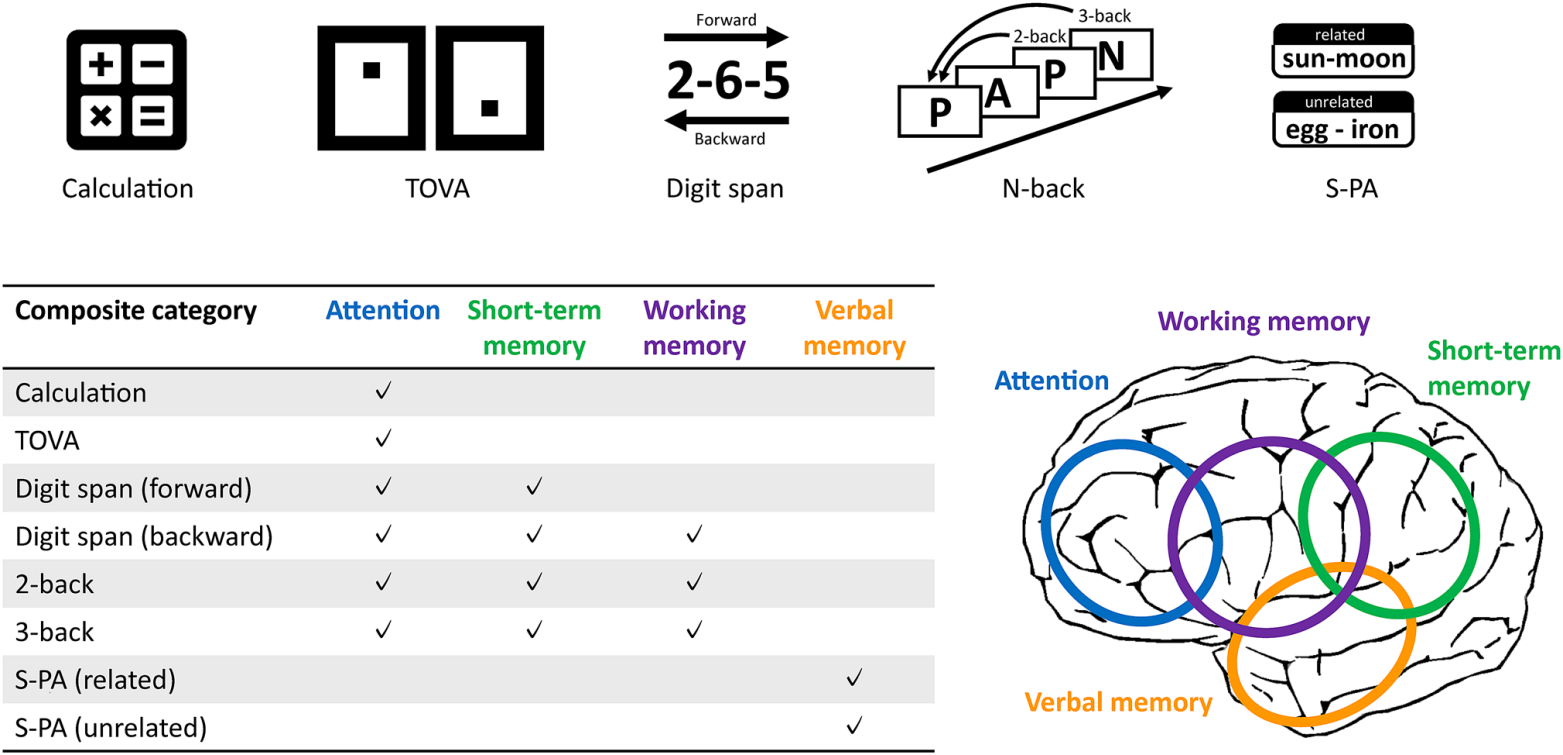
Cognitive tests and the corresponding brain functions. Eight cognitive tests derived from 5 tasks were used in this study. Four composite categories were generated based on the function each cognitive test was designed to evaluate. The right lower figure illustrates the rough area of the epicenter of each function. *TOVA* The Test of Variables of Attention, *S-PA* Standard verbal Paired Associate Learning Test

Feedback was delivered to each subject auditorily via earphones while listening to classical music, “Air on the G String” (Fig. 1). In NF, white noise was superimposed such that the noise level inversely correlated with nAP. Along with the sigmoid function, the volume of white noise was set to zero and the maximum when nAP was 2 and -2, respectively. The maximum volume levels of music and white noise were approximately 60–70 and 60 dB, respectively. The subject was instructed to minimize the noise level by increasing alpha-power as much as possible. In RF, the volume of white noise superimposed onto music varied in the same range as NF according to the randomly mixed EEG data of healthy volunteers. Therefore, subjects listened to a prerecorded sound during RF, while the recoding of EEG was performed using ALPHA SWITCH (Fig. 2).

### Data Analysis of Neurofeedback

**Fig. 3:**
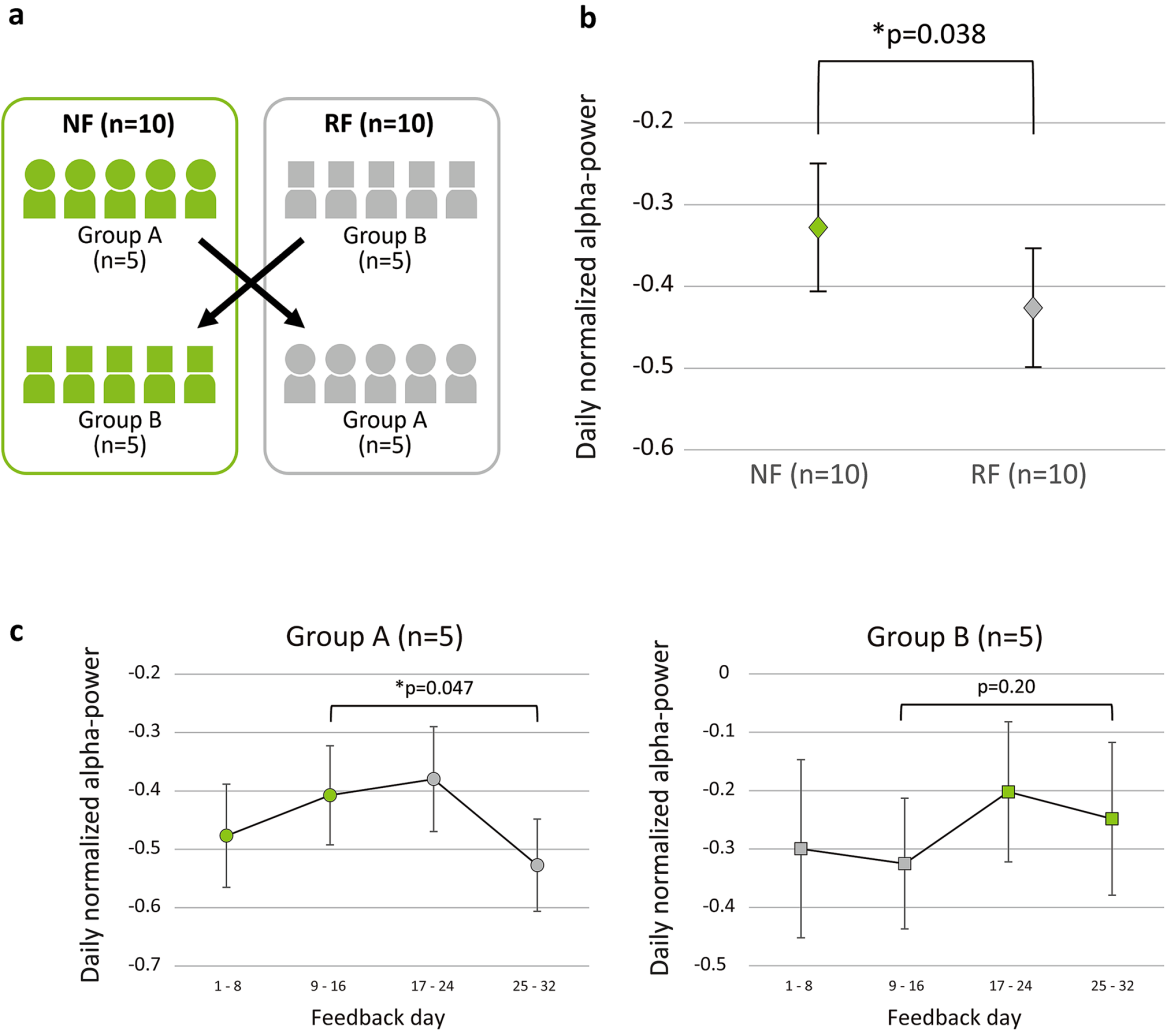
Comparison of the normalized alpha-power between normal and random neurofeedback. A schema of comparison in a crossover design (**a**). Yellow-green and gray colors refer to each subject in normal and random neurofeedback (NF and RF), respectively. Comparison between NF and RF regardless of the groups (**b**). The daily normalized alpha-power in NF was significantly higher than that in RF (*p* = 0.0383). The time courses of the daily normalized alpha-power are shown separately for group A and B (**c**). Group A showed a slight increase in NF (yellow-green) and a decrease in RF periods (gray). The daily normalized alpha-power of the late section was significantly higher in NF than RF period (*p* = 0.047)

Data recorded during feedback sessions were extracted from the tablet and analyzed offline using custom-made Matlab (The Mathworks, Natick, USA) programs. Previous studies on alpha wave neurofeedback reported that it took two weeks or 8 days of feedback before a significant difference was noted between the feedback and control groups (Lau-Zhu et al., 2019). Based on these findings, the impact of neurofeedback in the present study was assumed to be detectable in the late half of each feedback period. Each feedback period was divided into an early section (1–8 days) and late section (9–16 days) and the following analysis was applied in the late section. Time-averaged nAPs were averaged among three sessions in each day, yielding daily nAP. Since noisy nAPs were not omitted during feedback sessions, we excluded the maximum and minimum as outliers from the 8 daily nAPs of each section. Then, the average of 6 daily nAPs, which represented the section, was calculated. The daily nAPs of the late section of the first period in group A and the late section of the second period in group B were combined to represent the daily nAPs of NF across the groups. Similarly, the daily nAPs of the late section of the second period in group A and the late section of the first period in group B represented the daily nAPs of RF across the groups (Fig. 3a). The daily nAPs of NF and RF were statistically compared using a paired *t*-test (α = 0.05). Before the comparison, the normality of the distribution was tested using the Shapiro–Wilk test.

In addition to the above analysis, a robust regression analysis of 16 daily nAPs in the NF period was performed for each subject. Subjects were classified into responders or non-responders depending on whether the regression coefficient was positive or not, respectively. We then examined the association between responsiveness of NF and cognitive test scores, as described below.

### Cognitive Tests

Cognitive functions were evaluated three times; before, between, and after each feedback period. Each subject was assessed using the digit span test (DST), standard verbal paired-associate learning test (S-PA), simple calculation task (SCT), N-back test (NBT), and Test of Variables of Attention (TOVA) in this order. The total duration of all cognitive tasks was approximately 45 min.

In DST, the subject listened to a series of digits that were read out by an examiner at a pace of one digit per second and was asked to repeat them in the same (forward DST) or reverse order (backward DST) as presented. The digit number was increased one by one before two consecutive errors were made. The maximum digit number was the score of each task (Giofre et al., 2016; Woods et al., 2011).

In S-PA, the subject listened to and remembered a series of word pairs that were read out by an examiner at a pace of one word per second. The examiner then read out one word of each pair and the subject was asked to respond with the other word of the pair. S-PA consists of three batteries, each of which has two sets of ten pairs of Japanese words with and without a semantic relationship. The score of this task was expressed as the total number of correctly recalled words in three trials with the same set (Koike & Sugishita, 2011). To avoid the learning effect, different batteries were used in the three evaluation periods.

In SCT, 250 questions consisting of four arithmetic operations using single-digit numbers were presented and the subject was asked to solve as many questions as possible in three minutes. The score of this task was the number of correct answers.

In NBT (Owen et al., 2005), 8 letters of the alphabet were presented by a PC using the open-source Matlab script, ‘N-Back-for-Matlab’ (Layden, 2018), in a random order with an interval of 1.8 or 2.0 s for two- and three-back tests, respectively. The subject was asked to push a key when the presented letter was the same as that presented two (two-back test) or three trials (three-back test) earlier. The two-back test was performed using 20 letters as a rehearsal. The two- and three-back tests were then performed using 40 letters each. The score of this task was provided by the above mentioned Matlab script, as described previously (Layden, 2018).

In TOVA, two types of figures, each of which has a black dot on the upper or lower sides of a white square, were displayed on a monitor at the same frequency for 20 min. The subject was asked to push a button when a figure with a black dot on the upper side was presented. The TOVA score was estimated from the TOVA database based on the response speed, accuracy, and age (Forbes, 1998; Greenberg & Waldman, 1993).

### Data Analysis of Cognitive Test Scores

Since each subject performed the cognitive tests before and after the first neurofeedback period and after the second feedback period, we collected 30 scores from 10 subject regarding each cognitive test. To investigate the effects of neurofeedback on each test, the score of each subject was normalized using the mean and standard deviation of the 30 scores. Differences in scores between before and after feedback were considered to reflect the effects of neurofeedback on each test.

The tests of cognitive functions used in the present study were categorized into 4 composite categories (attention, short-term memory, working memory, and verbal memory), as shown in Fig. 2, by referring to the literature on methods to evaluate cognitive functions (Baddeley, 1992; Cullum, 1998; Kane et al., 2007; Owen et al., 2005; Reynolds, 1997) and instructions on sub-items of the established tests of cognitive functions, such as WAIS (D Wechsler, 1997; David Wechsler, 2008), WMS (David Wechsler, 1987), WISC (David Wechsler, 1991), the Standard Verbal Paired-Associate Learning Test (JSfHB, 2011), N-back test (Owen et al., 2005), and TOVA (Greenberg & Waldman, 1993).

The mean z-score among the cognitive tests was calculated in each composite category (Bird et al., 2019) and the scores of responders and non-responders were statistically compared using the Student’s *t*-test (α = 0.1). Prior to comparisons, the normality of the distribution was tested using the Shapiro–Wilk test (Royston, 1992).

## Results

### Verification of Alpha Wave Neurofeedback

nAP is normalized alpha-power calculated every three seconds based on the corresponding calibration data. By averaging all the time-series nAPs of three sessions in every feedback day, daily nAPs were obtained. According to the crossover design of the present study, the daily nAP of NF and RF across groups A and B were compared (Fig. 3a). Before the comparison, the normality of the distribution of each nAP was confirmed. As shown in Fig. 3b, the nAP of the late section was significantly higher in NF (*p* = 0.0383), which suggested that constant neurofeedback increased the inducibility of the alpha wave in each session.

A crossover design generally has a washout period between the first and second interventions to avoid type 1 errors due to the carryover effect. However, the appropriate interval of the washout period is unknown in neurofeedback studies. In the present study, the first and second neurofeedback periods were separated by at least two days, which may not have been sufficient to eliminate the sustained effect of the first neurofeedback session. To elucidate the impact of the sustained effect, a longitudinal analysis of nAPs was performed in each group (Fig. 3c). Group A showed a slight increase in NF and a slight decrease in RF. In group A, nAP of the late section was significantly higher in NF than in RF (*p* = 0.047). On the other hand, group B showed a slight decrease in both periods. No significant differences were observed in the nAPs of the late section between RF and NF; however, the latter was slightly higher.

### Changes in Cognitive Function

**Fig. 4:**
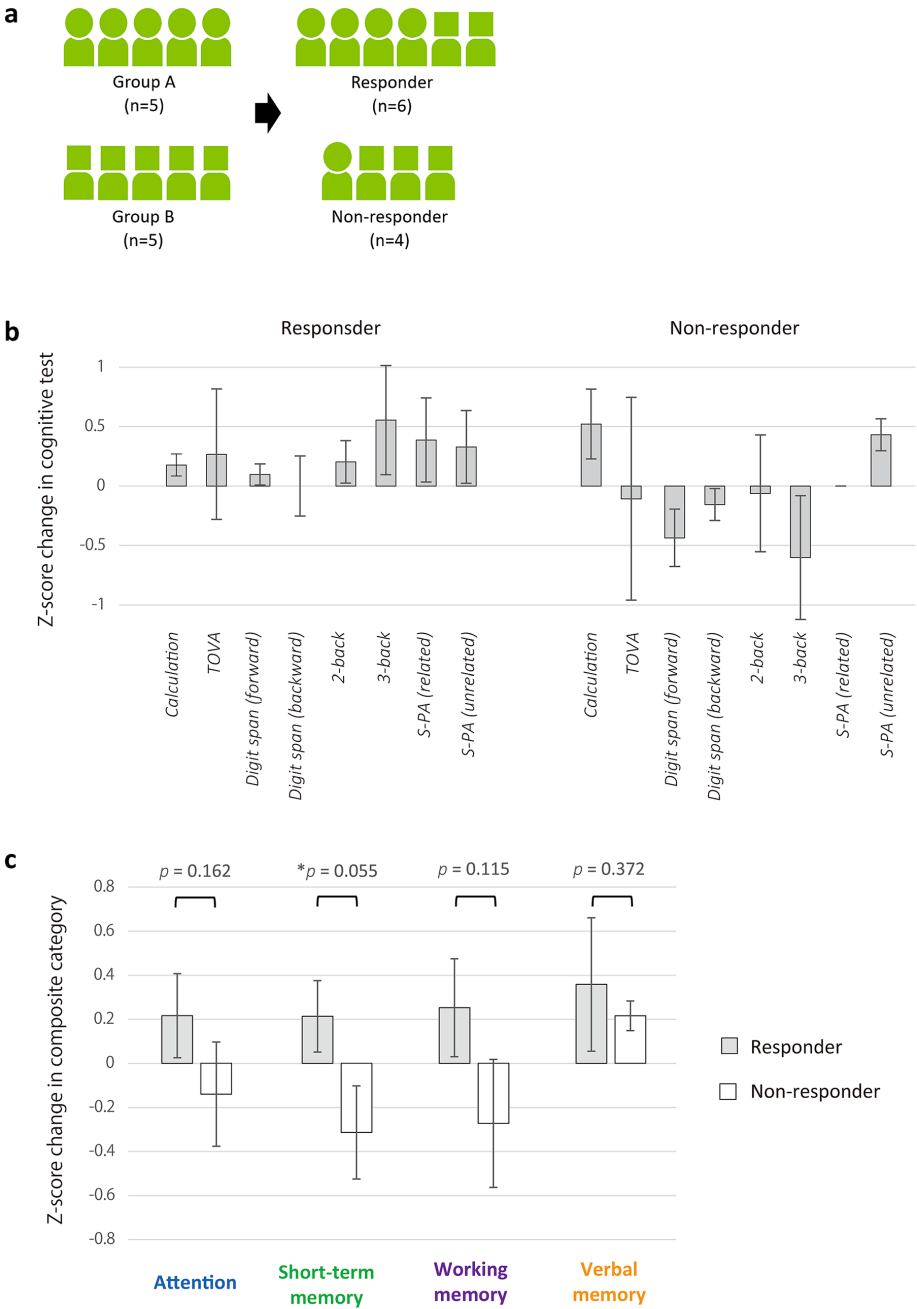
Comparison of changes in cognitive functions between responders and non-responders. Classification based on robust regression analysis revealed 6 responders and 4 non-responders (**a**). Changes in normalized cognitive test scores were compared between responders and non-responders (**b**). In each test, the change in the z-score was not significantly different between the two groups. A decrease was not observed in responders, whereas 5 out of 8 tests showed a decrease in non-responders. Changes in mean z-scores of each composite category were compared between responders and non-responders (**c**). In the four composite categories, only short-term memory showed significant difference

To evaluate cognitive test scores in association with responsiveness to neurofeedback, subjects were classified into responders and non-responders based on the results of a robust regression analysis of the time course of daily nAPs, yielding 6 responders (group A: 4, group B: 2) and 4 non-responders (group A: 1, group B: 3) (Fig. 4a).

Changes in normalized cognitive test scores were compared between responders and non-responders (Fig. 4b). In each test, the z-score change was not significantly different between the two groups. A decrease was not observed in responders, whereas five out of eight tests showed a decrease in non-responders, suggesting that alpha wave neurofeedback improved a diverse spectrum of brain functions after 16 days of consecutive sessions.

Changes in the mean z-scores of each composite category were then compared between responders and non-responders (Fig. 4c). Prior to each comparison, the normality of the distribution of the scores of corresponding composite categories were confirmed in each group, except for verbal memory. In the four composite categories, short-term memory showed a significant improvement (*p* = 0.055).

## Discussion

In the present study, ten subjects underwent NF and RF constantly for 32 days over more than two months. A crossover analysis revealed that normalized alpha-power was significantly higher in NF than in RF. This result demonstrated that constant auditory neurofeedback using music enabled subjects to easily induce the alpha wave, which was the primary end point of the present study. Auditory alpha neurofeedback was easy to continue and a promising protocol that warrants further development.

Changes in cognitive functions by neurofeedback were investigated as a secondary endpoint. A composite category-based analysis revealed that the degree of improvement in short-term memory was significantly higher in responders than in non-responders. This result suggested that constant auditory alpha wave neurofeedback reinforced short-term memory.

Only a limited number of previous studies on music-based auditory alpha neurofeedback have been reported. Alexander et al. conducted a study using a single session of musical alpha neurofeedback for 16 subjects and reported improvement in their mental status (Alexander, 2018). Ramirez et al. showed that 5 weeks of alpha and beta neurofeedback training improved depressive condition in 6 elderly people (Ramirez et al., 2015). These studies successfully suggested neuro-modulatory effect of musical auditory alpha neurofeedback. The present study is of notable significance in that it validated the effectiveness of musical auditory alpha neurofeedback by adopting longer-term crossover design and showing an increase in alpha power as well as improvement in cognitive function.

### Time Course of Effects of Auditory Alpha Wave Neurofeedback

The present results confirmed that auditory neurofeedback with a wearable EEG device for 16 days in 4–6 weeks enabled the augmentation of alpha-power. The majority of previous studies adopted a neurofeedback period ranging from a single session (Hanslmayr et al., 2005) to one week (Zoefel et al., 2011). With the aim of developing a neurofeedback system that may be constantly and casually performed, we validated a long-term neurofeedback system using the wearable EEG device. The entire feedback period consisted of NF and RF periods of 4–6 weeks each, lasting more than two months, which is one of the most constant protocols used in long-term neurofeedback studies. Daily normalized alpha-power in the NF period of group A was higher in the second two weeks than in the first, suggesting that a period of at least two weeks of neurofeedback is needed for effective alpha wave control using wearable EEG devices.

On the other hand, the longitudinal analysis of normalized alpha-power in each group suggested a sustained effect presumably due to preceding feedback. In group A, in which NF preceded RF, daily normalized alpha-power further increased in the early section of RF and decreased in the late section. This was assumed to be a sustained effect of a strategy developed for alpha wave control in preceding NF. In contrast, in group B in which RF preceded NF, the increase observed in daily normalized alpha-power was not significant. The unexpected decrease in the alpha power observed during NF sessions in group B might be caused by decline in subjects’ motivation due to long-term feedback over 2 months. We used the same music throughout all sessions across the study, which could have negatively affected the result. Changing music in the course of feedback sessions or using relaxing one could be useful to maintain the neurofeedback effect longer (Phneah & Nisar, 2017). Also, we could speculate that a wrong strategy developed during the preceding RF period might have canceled the effects of NF through the entire period. By taking the opposing effects of both NF and RF into consideration, the effects of one-month-long neurofeedback, both positively and negatively, may have prolonged it for more than two weeks.

### Relationship Between Alpha Wave Control and Cognitive Functions

Controversy surrounds the effects of alpha neurofeedback (Rogala et al., 2016) on cognitive functions due to negative findings (Naas et al., 2019). In the present study, the degree of improvement in short-term memory was higher in responders than in non-responders, which is consistent with previous findings showing an improvement in short-term memory in association with alpha wave augmentation (Hsueh et al., 2016; Klimesch, 2012; Klimesch et al., 2006; Zoefel et al., 2011).

Although the theta wave has been a major topic of interest in terms of the relationship between memory and EEG, the alpha wave also plays a pivotal role in memory and a wide range of cognitive functions (Jensen et al., 2002; Klimesch, 2012). Klimesch et al. reported that the alpha wave was crucially involved in the inhibition and timing of neuronal activation, and provides accessibility to stored knowledge through fundamental functions of attention, such as suppression and selection. The improvement in short-term memory associated with an increased alpha wave in the present study appears to be consistent with these assumed roles of the alpha wave.

There are various advantages to using the alpha wave in long-term neurofeedback, which is supposed to be performed in daily life. The alpha wave is the most robust brain activity recorded by scalp EEG across a wide age range, from childhood to old age (Smit et al., 2012). A reliable alpha wave recording is easily verified by opening and closing the eyes. Due to the universality and manageability of the alpha wave, the finding of improved short-term memory appears to be promising, which encourages the further development of alpha wave neurofeedback systems.

It is important to note that the improvement observed in the score of the working memory category was slightly better in responders than in non-responders (*p* = 0.115). Working memory may improve with enhancements in short-term memory because visual and auditory short-term memory constitute the core of Baddeley’s model of working memory (Baddeley, 1992). The lack of a significant improvement in working memory may have been due to the effect size and number of subjects in this study being selected to primarily validate the influence of the alpha wave neurofeedback system, which was the primary purpose of the present study.

### Advantages of the Wearable Neurofeedback System in the Present Study

While an increasing number of studies on alpha wave neurofeedback using high-performance clinical EEG machines have been reported (Guez et al., 2015; Navarro Gil et al., 2018; Zoefel et al., 2011), few have employed a neurofeedback system using portable EEG devices (Stopczynski et al., 2014; Wei et al., 2017). Further studies are needed to establish an easy-to-use neurofeedback system utilizing portable, particularly wireless, and wearable EEG devices in order to promote the generalized use of neurofeedback in daily life.

The wearable device used in the present study has excellent device mobility and sufficient system specification. Furthermore, the software that automatically performs the analysis and feedback may be easily incorporated into a tablet device by each user. These characteristics pair perfectly with the musical auditory alpha wave feedback protocol used in the present study. It is encouraging that the combination achieved alpha wave augmentation and improved short-term memory. This may be helpful for children with ADHD and elderly individuals with mild cognitive impairment, who may require constant and long-term neurocognitive rehabilitation.

### Limitations

It was difficult to recruit subjects who were able to fully cooperate such a constant and long-term protocol in the controlled condition, resulting in the small sample size of the present study. This preliminary result needs to be further validated in a larger study.

Also, we validated the feedback protocol by incorporating normal and random feedback sessions in a crossover design, without directly comparing each session to a control session in which subjects just listen to a music. This could be a confounding factor, which should be addressed in future study.

Since cognitive function tests were scheduled in the everyday life of subjects, it was difficult to exclude the effects of the physical and mental exhaustion of each day on the results of the tests. Therefore, the results of the cognitive tests included not only the effects of neurofeedback itself, but also considerable errors from the fluctuating state of mind of each subject. In other words, the present study reflects real-world circumstances, which is an important element in validating a system intended to be used in daily life.

## Conclusion

In healthy subjects, an auditory alpha wave neurofeedback system with a novel music-based protocol using an easy-to-use wearable EEG device was validated. A crossover analysis revealed that normalized alpha-power was significantly higher in NF than in RF, showing that constant neurofeedback with the system allowed subjects to easily induce the alpha wave. A composite category-based analysis of cognitive functions revealed that the degree of improvement in short-term memory was higher in responders than in non-responders. The present study adopted one of the most constant protocols used in previous long-term neurofeedback studies and achieved successful neurofeedback in terms of alpha wave control and cognitive function. This is encouraging for children with ADHD and elderly individuals with mild cognitive impairment, who may require constant and long-term neurocognitive rehabilitation.

## Data Availability

Due to the nature of this research, participants of this study did not agree for their data to be shared publicly, so supporting data is not available.
